# Self-Assessed Personality Traits and Adherence to the COVID-19 Lockdown

**DOI:** 10.3390/ijerph20010521

**Published:** 2022-12-28

**Authors:** Jakub Jan Rojek, Przemysław Waszak, Ilona Bidzan-Bluma, Aleksandra Sanewska, Joanna Stępień, Tomasz Michalski, Liliana Lorettu, Roberta Meloni, Che-Sheng Chu, Myriam Abboud, Jakub Grabowski

**Affiliations:** 1 Adult Psychiatry Scientific Circle, Department of Developmental Psychiatry, Psychotic and Geriatric Disorders, Medical University of Gdańsk, 80-282 Gdańsk, Poland; 2 Departament of Hygiene and Epidemiology, Medical University of Gdańsk, 80-210 Gdańsk, Poland; 3 Departament of Psychology, Gdańsk University of Physical Education and Sport, 80-336 Gdansk, Poland; 4 Institute of Psychology, Faculty of Social Sciences, University of Gdansk, 80-309 Gdansk, Poland; 5 Department of Socio-Economic Geography, Faculty of Social Sciences, University of Gdańsk, 80-309 Gdańsk, Poland; 6 Department of Regional Development, Faculty of Social Sciences, University of Gdańsk, 80-309 Gdańsk, Poland; 7 Psychiatric Clinic, Department of Medical, Surgical and Experimental Sciences, University of Sassari, 07100 Sassari, Italy; 8 University of Sassari, 07100 Sassari, Italy; 9 Department of Psychiatry, Kaohsiung Veterans General Hospital, Kaohsiung 813, Taiwan; 10 Center for Geriatrics and Gerontology, Kaohsiung Veterans General Hospital, Kaohsiung 813, Taiwan; 11 Graduate Institute of Medicine, College of Medicine, Kaohsiung Medical University, Kaohsiung 807, Taiwan; 12 Department of Natural Science and Public Health, Zayed University, Dubai P.O. Box 19282, United Arab Emirates; 13 Department of Developmental Psychiatry, Psychotic and Geriatric Disorders, Medical University of Gdańsk, 80-282 Gdańsk, Poland

**Keywords:** pandemic, isolation, quarantine, cultural differences, optimism, openness

## Abstract

**Introduction:** The COVID-19 pandemic, caused by the SARS-CoV-2 coronavirus, has forced all countries affected by it to introduce quarantine and isolation to prevent the spread of the virus, as well as masking and distancing. Not everyone is equally willing to follow the rules related to limit the extent of the coronavirus epidemic. This might be connected with personality traits, especially openness, positive attitude, and optimism. **Materials and Methods**: An online survey was created and completed by participants in April–May 2020. Self-assessment of personality traits and adherence to lockdown recommendations were assessed. A total of 7404 participants took part in the study, mainly from Poland (83.6%) and Italy (12.7%). Univariate and multivariate regression analysis was performed. **Results:** The participants were divided into groups depending on the degree of compliance with the lockdown rules. In the multivariate analysis, variables that increased the odds for stricter lockdown compliance were temporary work suspension OR 1.27 (95% CI 1.10–1.48), income level “we can’t handle this situation” OR 1.67 (95%CI 1.20–2.33), and junior high school education OR 1.68 (95% CI 1.13–2.50). Other significant factors included age and place of residence. Each point of self-assessed sociability OR 1.07 (95% CI 1.00–1.13) also increased the likelihood of adhering to lockdown rules. **Conclusions:** Taking the basic demographic characteristics as well as working and health environment conditions traits into account may be helpful when forecasting epidemiological compliance during a pandemic, as well as in other public health tasks. The key role of self-assessed personality traits was not confirmed in this study. Reliability of the results is limited by significant disproportions in the size of the study groups.

## 1. Introduction

Infectious diseases have accompanied people for centuries; possibly, they are the oldest known to mankind [1]. Besides a direct impact on the somatic state, infectious diseases also have a psychological impact [2] and an impact on mental health [3,4,5,6].

In the midst of the COVID 19 pandemic, people became much more aware of their own mortality [7]. Such a situation requires the individual to react and find a coping strategy. This, in turn, can take various directions, including either avoiding or facing adversities—which are based on genetic predisposition, early life experiences, and gene-environmental interactions—and may be modified as a result of interventions [8]. The modifiability of the approach to a pandemic makes governments take action to combat infectious diseases in political systems in the international arena.

### 1.1. The Characteristics Studied

Despite the development of medicine and the introduction of new therapeutic agents in the treatment of infectious diseases, the success of the therapeutic process is invariably influenced by human character traits. Compliance with top-down pandemic restrictions may be related to such personality traits [9].

Optimism is defined as the expectation of favorable events in the future [10]. It is a personality trait associated with a positive health impact, regardless of the features included in the model describing the main personality traits, or the so-called Big Five [11]. The situation related to the COVID-19 pandemic is indisputably one that has the characteristics of a stressor that needs to be addressed and requires attempts at coping mentally. Benzel [12], Bidzan-Bluma et al. [13], and Stueck [14] found that a positive attitude and optimism are supposed to strengthen coping with the pandemic.

Sociability can be defined as an individual’s tendency to participate in non-aggressive activities with other similar individuals (conspecifics) [15]. It is a complex multi-factor feature, and its role in relation to social isolation is underestimated

Openness is one of the features that make up the Big Five [16]. It is a complex feature [17], which makes it difficult to study it as a whole without breaking it down into its individual aspects.

So far, the relationships between stress and sociability and demographic data have been studied in the context of the limitations of the COVID-19 pandemic [18]. There is a lack of reports of an inverse relationship, i.e., the effect of traits on adherence. In view of the demonstrated links between the features and pro-health behaviors, it seems reasonable to look at the above-mentioned features in the context of compliance with the recommendations for beneficial health decisions that the lockdown was supposed to bring in each country.

### 1.2. Prevention of Infection Transmission

The methods of preventing spreading contagion have been similar over the centuries—quarantine and isolation. However, they are related to ethical and legal problems, such as limiting the freedom of the individual for the common good. Our previous studies on the subject showed that applying similar prevention principles may not bring the expected benefits for a given population [19]. Additionally, if measures are intended to ease the burden on healthcare, they must take into account the needs of minorities [20].

### 1.3. Apart from Advantages, Quarantine also Has Disadvantages

The negative health consequences of the obligatory preventive measures concerning the current pandemic can be found in scientific publications, apart from non-professional literature. About six months after the appearance of SARS-CoV-2, You et al. [21] conducted a study. It reported that forced quarantine is related to discrimination, mental discomfort, and thoughts of suicide or self-harm. Furthermore, a study published more than a year after the appearance of SARS-CoV-2 reported the occurrence of depression and anxiety symptoms in, respectively, 26.47% and 70.78% of 1160 people surveyed during quarantine, which is a higher percentage than the frequency of these disorders in the general population [22].

Chu et al. [23] reported the social consequences of quarantine, such as psychological discomfort, economic challenges, inconvenient access to medical care, problems related to the education system, and the intensification of violence. The above findings were common to previous quarantine times in previous epidemics and during COVID-19.

### 1.4. Attitudes towards the Recommended Course of Action

People differ in their daily attitude toward not only the recommendations of societies and specialist groups, or to the recommendations of the rulers and authorities, but also toward other, informally prevailing social rules [24]. The approach to the applicable regulations is different based on complex multi-factor relations [25]. These dependencies are mediated by the perception of oneself, what happens to us, and the associations that COVID-19 causes [26,27]. Moreover, it may be a result of social dependencies, such as gender and marital status [28,29,30].

Attempts to explain the phenomenon of compliance with health-related behavior recommendations are also available in the literature as reports based on HBM models [9,31]. Although personality is a relatively consistent way of thinking, experiencing emotions, behavior, and controlling drives, an individual’s actions are always an individual matter.

### 1.5. Lockdown

Different countries almost concurrently introduced similar but not identical types of recommendations associated with diminished mobility [32]. The first so-called COVID-19 lockdown was established by the EU countries almost simultaneously, but its scope was varied [33]. Before the introduction of SARS-specific vaccines and drugs, lockdowns were the primary tool of prevention, and adherence to their rules was crucial to public health [34].

### 1.6. Aim of the Study

The aim of the study was to evaluate a possible association between the optimism or pessimism of the respondents and the compliance with sanitary rules determined by the government recommendations during the COVID-19 pandemic. Moreover, the relationship of another personality trait, sociability, was studied. Finally, the research took into consideration the differences between nationalities. The obtained results may be beneficial in considering the possible new ways of implementing the recommendations hereafter.

The key features in the study were optimism, pessimism, sociability, openness, and their relationship with compliance with the lockdown recommendations, as well as whether there is a relationship between the environmental conditions in which the respondents lived and their lifestyles, such as opportunity to work from home and being obligated to cope with one’s or one’s relative’s disease.

## 2. Materials and Methods

### 2.1. Participants

This a descriptive study, which was based on an online survey conducted during the first COVID-19 pandemic wave (April–May 2020). At that time, in many places around the world, especially in OECD countries, regulations of the so-called “lockdown” were implemented, and sanitary restrictions were imposed, e.g., closures, limitations, and warrants [35]. An original questionnaire was prepared, which was then sent online to potential participants. Then, the results were collected and statistically analyzed, and conclusions were drawn.

The survey was available in English, German, Polish, and Italian. The survey was distributed using the snowball sampling, a form of convenience sampling, and through the national and local media and websites, social media, university newsletters, etc.

Inclusion criteria were age > 18 years, a degree of understanding the language that allows completing the survey, and consent to participate in the study. Adult participants, after giving informed consent, could take part in the study. Apart from the lack or withdrawal of consent, there were no other exclusion criteria. The study complied with all guidelines and ethical standards for human online surveying, in accordance with the ethical principles of the Helsinki Declaration. Survey data were collected online using Google Forms (Google Inc., United States) and subsequently exported to Excel spreadsheets (Microsoft, United States).

The study was part of a larger project whose partial results on participants’ perceived stress have previously been published [19,20,24,32].

### 2.2. Measured Variables

All participants were asked about sociodemographic data, including their nationality, financial situation during the time of the pandemic, working style during the lockdown, and basic health information.

The personality part was based on a self-assessment questionnaire with five possible Likert-type scale responses [36]. The four personality traits measured were sociability, calmness, openness, and optimism. Each participant was asked to rate themselves for each of these traits on a scale of 1 (least subjective trait intensity) to 5 (highest subjective trait intensity). It was necessary to answer the personality questions in order to move on to the subsequent parts of the survey.

The next part of the survey concerned declarations related to compliance with the lockdown rules. There were 9 questions concerning mainly leaving home for various reasons (important situations, work, shopping, church, walk, public places, meeting with friends, training, going out with a pet/child). The participants were asked to rate how closely a sentence corresponded with their current life activity on a five-point scale: definitely agree (−2), mostly agree (−1), not applicable (0), rather disagree (1), and definitely disagree (2). The points helped to develop a scoring system. The points from all 9 questions were added together to form the final score. The maximum number of points it was possible to earn was 18, the minimum −18. We established −6 points (the median) as the cut-off point to divide the participants into roughly two equal groups. This resulted in creating more adhering (54%) and less adhering (46%) groups.

### 2.3. Statistical Analysis

Statistical analysis was performed using STATISTICA 10.0 software (StatSoft Inc., St. Tulsa, OK, United States). We used Cronbach’s alpha to measure the reliability and internal consistency of the scales used in the questionnaire (Supplementary Materials). Descriptive statistics were initially used to characterize the study population. Verification of whether the sample came from a normally distributed population was made using the Shapiro-Wilk test, while Levene’s (Brown-Forsythe) test was used to assess the equality of variances for a variable calculated for two or more groups. Depending on whether the variable met the normality condition, appropriate statistical tests were applied in further stages. For comparisons between two groups, the parametric t-test or non-parametric Mann—Whitney U-test was used. For Gaussian data, comparing several groups, we used the one-way ANOVA. If the result was significant for particular group differences, we ran a post hoc Scheffé’s test (to minimize the potential unequal sample size bias). To compare qualitative survey data, Pearson’s chi-squared test was used along with the calculation of observed frequencies (with appropriate Yates’ correction for small observed frequencies when necessary).

In the next step, univariate regression with effect sizes and R-square was calculated, and then finally stepwise multivariate logistic regression analysis was performed. The area under the curve (AUC), R-square, and F-value were calculated. Odds ratios were calculated with 95% confidence intervals. The significance levels for all analyses were *p* < 0.05 and *p* < 0.01.

## 3. Results

### 3.1. Basic Characteristics

The study involved 7393 participants, mainly from Poland (83.6%). Eleven respondents were excluded due to incomplete answers. Most of the participants were women (73.3%), and most respondents declared higher education (67.9%). Italian respondents constituted the second-largest group (12.7%), while small groups of respondents came from the United Arab Emirates (UAE), Taiwan, Germany, and Japan (Table 1). Most of the participants were young (< 44 years old), living in larger cities (> 150,000), mostly working from home (41.9%), and assessing their financial situation as good (58.4%) during the COVID-19 pandemic. Nearly a third of the study participants had chronic disease (31.7%) and remained mostly free from COVID-19 infection (Table 1). The raw Cronbach’s alpha for self-assessed personality traits was 0.61 (95% lower confidence limit = 0.54), and for lockdown compliance questions, it was 0.67 (95% lower confidence limit = 0.66). The exact alpha results for each item are given in the appendix to this manuscript (Table S1).

### 3.2. Self-Assessed Personality Traits

Women, Italians, people aged 55–64, divorced individuals, inhabitants of medium-sized cities, and people with vocational education had the highest values of self-assessed sociability in the analyzed period (Table 2). Men, Japanese, village inhabitants, elderly people aged 75–84, widows, and people with primary education level assessed their calmness as highest compared to other groups (Table 2). The participants with the highest ratings for their openness were women, Germans, people aged 75–84, divorced individuals, those with vocational education, and inhabitants of the largest cities (>500,000). Similarly, optimism was rated the highest by men, UAE citizens, people aged 75–84, widows, those with vocational education, and people from the largest cities (>500,000).

### 3.3. Logistic Regression

**Figure 1:**
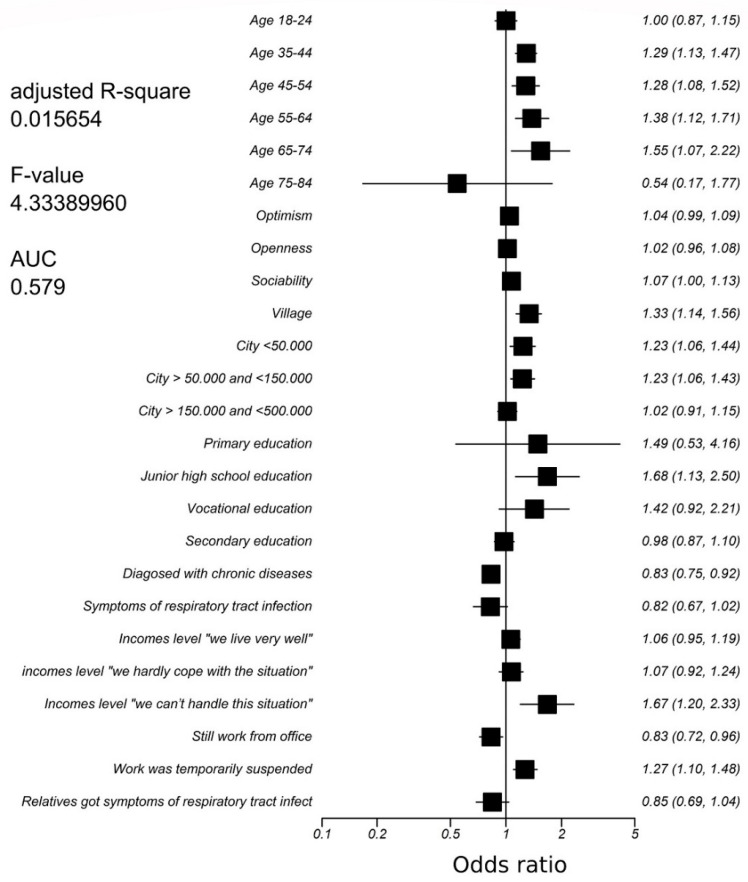
Results of multivariate logistic regression for stricter lockdown compliance.

In the one-way analysis, sociability, optimism, openness, age 55–64, living in a city of 50,000–150,000 citizens, not having chronic condition or symptoms of respiratory tract infection, not having relatives with such symptoms, and temporarily suspended work were statistically significantly associated with an increase in the odds for stricter adherence to lockdown rules (Table 3). On the contrary, living in a city of 150,000–500,000 or > 500,000, higher or secondary education, earning “fine” or “very well”, and working from the office were associated with less compliance to lockdown rules. In the final multivariate analysis (Figure 1), the variables that increased the odds for stricter lockdown compliance were temporary work suspension OR 1.27 (95% CI 1.10–1.48), income level “we can’t handle this situation” OR 1.67 (95% CI 1.20–2.33), and junior high school education OR 1.68 (95 CI 1.13–2.50). Other significant factors include age: 35–44 years OR 1.29 (95% CI 1.13–1.47), 45-54 years OR 1.28 (95% CI 1.08–1.52), 55–64 years OR 1.38 (95% CI 1.12–1.71), age 65–74 OR 1.55 (95% CI 1.07–2.22), and place of residence: village OR 1.33 (95% CI 1.14–1.56), city < 50,000 OR 1.23 (95% CI 1.06–1.43), city 50–150,000 OR 1.23 (95% CI 1.06–1.43). Each point of self-assessed sociability OR 1.07 (95% CI 1.00–1.13) also increased the likelihood of following lockdown rules.

Significant factors that reduced the odds of lockdown compliance were working from the workplace during the study OR 0.83 (95%CI 0.72–0.96) and being diagnosed with chronic disease OR 0.83 (95%CI 0.75–0.92). The total area under the ROC curve (AUC) for the model was 0.579 (95% confidence interval 0.568 to 0.591) and standard error 0.00664.

## 4. Discussion

The approach to preventing the spread of the pandemic has varied between countries, and people who have individual differences in compliance with the quarantine rules [37].

Our results indicate that there was no increased likelihood of optimistic individuals obeying lockdown rules. This is opposite to the fact that optimism is one of the factors associated with good health [38,39]. People with an optimistic outlook engage and persist in positive health behaviors [38], and it seems that in a pandemic situation, this factor may be helpful in sticking to health recommendations. However, there are numerous conspiracy theories regarding the COVID 19 epidemic that could affect the general perception of the regulations, offset the optimism effect, disturb the individual’s judgment, leading to anti-health behavior.

Optimism is also conducive to openness to changes, and therefore reinforces adaptation skills [40], which could have led to better adherence to the COVID-19 lockdown, but results of our research do not confirm this. The optimist believes that he has an influence on different events. He is also convinced that his behavior determines the situations that will occur in the future [41]. Because of this, following the recommendations will positively affect his health and that of his relatives. Similar results were shown by Woodland et al. [42], who reported that optimism has a crucial role in adherence to the regulations by family members in the examined group. Despite that, the association could not be so simple, and further research is needed. In addition, not having any relative with respiratory infection at the time of the study was shown to be associated with stricter lockdown compliance. This can be explained by the fact that in the case of falling ill in the family, someone had to take over the duties of the sick person (such as shopping, walking the dog, and religious practices), which could be associated with a violation of the restrictions. Furthermore, Smith et al. [43] reported that adherence is related to the opportunity to get help from someone outside the household.

Among the respondents, men were more likely to break the restrictions than women, which was also demonstrated by Pollak et al. [44] or Smith et al. [43], who reported that the surveyed men were more likely to leave their households despite contact with household members with confirmed COVID infection. These results are interesting because previous research reports regarding the impact of gender on health belief behaviors are ambiguous [45]. This may also be due to personal value systems as well as demographic factors [45,46]).

The study showed that the ability to work remotely is one of the factors that allows for compliance with the restrictions. Recommended by the WHO, it is one of the basic methods of preventing the spread of infection [47]. It seems intuitive that people deprived of this possibility will not decide to leave their jobs in the face of an uncertain economic situation in the world. Having a chronic disease was another factor. This may be due to the fact that people with chronic disease are more likely to get sick and may also have had a worse course of coronavirus (with serious diseases of the cardiovascular system, such as heart failure, coronary artery disease, cardiomyopathies, or cancer, chronic kidney disease, chronic respiratory disease, etc.) [48,49]. It was also publicized by the media.

The trait of neuroticism (calm) was not statistically significant. Similar [50,51] as well as opposite [52,53] conclusions can be found in the literature.

It is worth noting that optimistic attitude, openness, optimism and sociability showed no effect on the likelihood of adherence to isolation rules during the COVID-19 pandemic. This may have to do with the fact that people who are characterized by these traits do not experience high levels of anxiety in any situation, which certainly induces isolation adherence during a pandemic. This is supported, among other things, by the findings of McColl et al. [54], indicating that unrealistic optimism is negatively associated with the adoption of protective behaviors, which is worrying, given that these preventive measures are critical in tackling the spread and health burden of COVID-19.

On the one hand, analyses showed that variables that increase the odds of exacerbating the restriction are temporary suspension from work, diagnosis of chronic diseases, symptoms of respiratory tract infections, and the presence of symptoms of respiratory tract infections in loved ones. The existence of such diseases, as confirmed by many researchers, is related to a more severe course of the disease known as COVID-19, and even death [55]. Not surprisingly, chronic illness and/or symptoms of respiratory infection increase adherence to isolation during the pandemic.

On the other hand, the obligation to work in an office due to the need to comply with the employer’s requirements and the inability to influence this decreased adherence to the recommendations, as also indicated by Wnuk et al. [56], who also emphasized that perceived personal threat and lack of personal control are significantly and positively related to the acceptance of surveillance technologies.

According to Tseng et al., quarantine cannot be effective if it is disregarded by the population [57].

### 4.1. Limitations of the Current Study

The main limitation of the present study is the highly unequal sample—with the Polish residents being the most numerous group, followed by Italians. Other countries were represented by far fewer respondents. Moreover, each country was in a different stage of the pandemic (number of new cases and deaths) when the survey was conducted, which also could have interfered with social behaviors and the response to restrictions.

The study implemented the self-description of some personality traits, such as optimism, instead of psychological tests examining the respondents’ personality, which may be a further limitation. Furthermore, self-reported adherence measures have been implemented in the study and might be susceptible to social desirability bias [58]. Additionally, women are overrepresented in the sample. Another concern is that recognizing a trait such as sociability may be of limited value because the reports in the literature are usually based on the subjective assumptions of the respondents [59]. It is worth noting that most of the respondents (81.1% of the included group) were younger (<44 years old) and probably more likely to respond to an Internet survey. The only way to access the questionnaire is through the Internet. This situation resulted in a smaller group (18.2% of the included group) of elderly (> 44 years old) respondents.

We expanded the statistical analysis in an effort to make our study more substantial. It is important to keep in mind that the effect sizes provided were very small. It is also challenging to extrapolate the reported results for wider use in practice, despite statistical significance. There was a risk of confounding factors as well. The main alpha values for major scales used in the tests were 0.61 and 0.67, which should also be considered unsatisfactory. Using the Spearman–Brown prediction formula, we may predict the reliability of a psychometric test after changing the test length. Just by adding two questions to each scale, we would obtain an alpha value greater than 0.7, and when doubling the number of questions, we would obtain alpha values of 0.76 and 0.80, respectively. These conclusions should be kept in mind when constructing future research on a similar topic.

### 4.2. Research Strengths

The study’s strengths include the study of participants during the first wave of the pandemic, the large group of respondents, and the remote form of the study, which provided participants with protection against possible infection. In addition, people answering remotely may have felt more comfortable, being in a familiar environment, and the questionnaire was fully anonymous, which supported honesty when answering the questions.

## 5. Conclusions

In this work, an assessment of the influence of some personality traits, such as optimism or pessimism, on adherence to the Covid-19 lockdown was conducted. Some variables such as demographic affected results of the study.

It is worth noting that an optimism bias, openness, optimism, and sociability did not demonstrate the influence on the likelihood of obeying lockdown rules during COVID 19 pandemic. On the one hand, the analyses showed that the variables that increase the odds for stricter lockdown compliance are temporary work suspension, being diagnosed with chronic diseases, symptoms of respiratory tract infection, and relatives with symptoms of respiratory tract infection. On the other hand, obligation of working from office and age over 75 and below 84 years decrease compliance.

The research results may be beneficial for psychological support because they show what to look for and what skills are worth developing in people who have experienced a particularly negative impact of the pandemic on their emotional functioning.

## Figures and Tables

**Table 1:** Characteristics of the study participants.

	Count	Percent
**Age**		
18–24	2130	28.6
25–34	2365	31.8
35–44	1542	20.7
45–54	757	10.2
55–64	447	6.0
65–74	133	1.8
75–84	14	0.2
**Gender**		
male	1858	25.0
female	5456	73.3
**Martial status**		
single	3092	41.5
married/long term relationship	3916	52.6
divorced	310	4.2
widow	75	1.0
**Education**		
higher	5054	67.9
secondary	2125	28.6
vocational	89	1.2
junior high school	111	1.5
primary education	15	0.2
**Country**		
Poland	6221	83.6
Italy	944	12.7
UAE	54	0.7
Taiwan	45	0.6
Germany	31	0.4
Japan	45	0.6
other Europe	46	0.6
other Asia	13	0.2
other	5	0.1
**City size**		
city with between 50,000 and 150,000 citizens	1018	13.7
city with under 50,000 citizens	905	12.2
city with between 150,000 and 500,000 citizens	2033	27.3
city with over 500,000 citizens	2505	33.7
village	929	12.5
**Household size (people)**		
1	842	11.3
2	2305	31.0
3	1752	23.5
4	1716	23.1
5 and more	773	10.4
**Pandemic working condition**		
Yes, I work from home	3118	41.9
No, I still work from office	1119	15.0
No, work was temporarily suspended	1093	14.7
Not applicable	2061	27.7
**Financial situation**		
With current incomes level we are doing fine	4348	58.4
With current incomes level we live very well	1739	23.4
With current incomes level we hardly cope with the situation	905	12.2
Refuse to answer	244	3.3
With current incomes level we can’t handle this situation	156	2.1
**Symptoms of respiratory tract infection at the moment?**		
no	6957	93.5
yes	437	5.9
**Diagnosed with Coronavirus disease?**		
no	7378	99.1
yes	16	0.2
**Has anybody from your relatives got symptoms of respiratory tract infection at the moment?**		
no	6928	93.1
yes	464	6.2
**Has anybody from your relatives been diagnosed with Coronavirus disease?**		
no	7243	97.3
yes	151	2.0
**Are you diagnosed with chronic diseases?**		
no	5034	67.6
yes	2360	31.7

UAE—United Arab Emirates.

**Table 2:** Results of self-assessment of personality traits.

	Sociability			Calmness			Openness			Optimism		
Gender	mean	*p*	SD	mean	*p*	SD	mean	*p*	SD	mean	*p*	SD
Male	3.52	#	1.08	3.62	#	1.03	3.17	#	1.15	3.49	*	1.09
Female	3.65	#	1.02	3.35	#	1.01	3.42	#	1.13	3.44	*	1.07
Age group												
18–24	3.54	#	1.06	3.34	#	1.04	3.24	#	1.14	3.26	#	1.06
25–34	3.57	#	1.06	3.40	#	1.02	3.37	#	1.14	3.41	#	1.08
35–44	3.61	#	1.02	3.44	*	1.00	3.42	#	1.12	3.57	#	1.05
45–54	3.80	#	1.03	3.52	#	1.01	3.49	#	1.14	3.66	#	1.05
55–64	3.81	#	0.95	3.64	#	1.01	3.36		1.14	3.76	#	1.05
65–74	3.80		0.90	3.66	*	1.07	3.50		1.14	3.75	#	1.05
75–84	4.29		0.83	4.00		0.96	4.21	*	1.25	3.79		1.31
Martial status												
single	3.53	#	1.07	3.41	#	1.04	3.22	#	1.15	3.31	#	1.07
married/long term relationship	3.67	#	1.02	3.42	*	1.01	3.44	#	1.13	3.53	#	1.07
divorced	3.76	#	1.02	3.55		0.96	3.59	#	1.12	3.78	#	0.99
widow	3.73		1.04	3.77	#	0.92	3.56		1.07	3.85	#	0.97
Education												
higher	3.59	#	1.03	3.43		1.02	3.36		1.13	3.48	#	1.06
secondary	3.64	*	1.06	3.41		1.03	3.33		1.16	3.38	#	1.09
vocational	3.99	#	0.95	3.54		1.02	3.57		1.12	3.83	#	1.09
junior high school	3.77		1.04	3.34		1.07	3.36		1.13	3.13	#	1.16
primary education	3.87		1.19	3.67		1.05	3.47		1.19	3.27		1.33
City size												
50,000–150,000 citizens	3.78	#	1.01	3.37		1.05	3.36		1.14	3.49		1.08
>50,000 citizens	3.57	#	1.05	3.46		1.03	3.29	*	1.15	3.41		1.08
150,000–500,000 citizens	3.54	#	1.05	3.42		1.03	3.36		1.13	3.44		1.08
>500,000 citizens	3.62	#	1.04	3.41		1.02	3.42	#	1.13	3.47		1.07
village	3.64		1.05	3.48		1.01	3.25	#	1.16	3.43		1.08

SD—standard deviation, * *p* ≤ 0.05; # *p* ≤ 0.01; UAE—United Arab Emirates.

**Table 3:** Univariate regression analysis for stricter lockdown compliance.

	Coefficient of Determination R^2^	Effect Size (f-Square)	Variable	Odds Ratio	Lower 95%CI	Upper 95%CI	*p*-Value
No of people in household	0.0001731	0.00017	-	1.001	1.000	1.003	0.081
Calmness	0.00001373	0.000014	-	1.007	0.963	1.053	0.750
**Sociability**	0.003699	0.0037	-	**1.125**	**1.076**	**1.176**	**0.000**
**Optimism**	0.002391	0.0024	-	**1.096**	**1.050**	**1.144**	**0.000**
**Openness**	0.001904	0.0019	-	**1.080**	**1.037**	**1.124**	**0.000**
Sex	0.004676	0.0047	male	1.834	0.473	7.115	0.380
			female	1.339	0.346	5.185	0.672
Martial status	0.0001217	0.00012	single	0.946	0.824	1.086	0.430
			married/long term relationship	0.951	0.830	1.090	0.468
			divorced	1.181	0.963	1.447	0.110
Have you been diagnosed with Coronavirus disease?	0.00001581	0.000016	no	0.918	0.562	1.499	0.733
Has anybody from your relatives been diagnosed with Coronavirus disease?	0.0003628	0.00036	no	0.874	0.744	1.027	0.102
Age	0.002466	0.0025	35–44	1.131	0.929	1.376	0.220
			25–34	0.896	0.740	1.083	0.255
			45–54	1.191	0.961	1.475	0.110
			**55**–**64**	**1.282**	**1.012**	**1.625**	**0.039**
			18–24	0.965	0.797	1.168	0.713
			75–84	0.484	0.179	1.310	0.153
City size	0.00203	0.002	**city 50,000**–**150,000 citizens**	**1.120**	**1.006**	**1.248**	**0.038**
			city > 50,000 citizens	1.041	0.930	1.165	0.484
			**city 150,000**–**500,000 citizens**	**0.869**	**0.799**	**0.945**	**0.001**
			**city > 500,000 citizens**	**0.853**	**0.788**	**0.923**	**0.000**
**Have you got symptoms of respiratory tract infection at the moment?**	0.00135	0.0014	**no**	**1.173**	**1.062**	**1.295**	**0.002**
**Education**	0.0007124	0.00071	**higher**	**0.762**	**0.601**	**0.965**	**0.024**
			**secondary**	**0.759**	**0.596**	**0.967**	**0.026**
			vocational	1.229	0.824	1.835	0.312
			junior high school	1.344	0.924	1.955	0.123
**Are you diagnosed with chronic diseases?**	0.001838	0.0018	**no**	**1.097**	**1.044**	**1.153**	**0.000**
**Financial situation**	0.0002943	0.00029	**With current incomes level we are doing fine**	**0.818**	**0.740**	**0.904**	**0.000**
			**With current incomes level we live very well**	**0.830**	**0.740**	**0.931**	**0.001**
			With current incomes level we hardly cope with the situation	0.918	0.802	1.049	0.209
			Refuse to answer	1.077	0.870	1.334	0.493
**Do you currently work from home?**	0.0005826	0.00058	Yes, I work from home	0.970	0.904	1.042	0.404
			**No, I still work from office**	**0.828**	**0.751**	**0.914**	**0.000**
			**No, work was temporarily suspended**	**1.265**	**1.147**	**1.395**	**0.000**
**Has anybody from your relatives got symptoms of respiratory tract infection at the moment?**	0.001152	0.0012	**no**	**1.153**	**1.048**	**1.270**	**0.004**

## Data Availability

Upon reasonable request.

## Supplementary Materials

The following supporting information can be downloaded at: https://www.mdpi.com/article/10.3390/ijerph20010521/s1.

## References

[1] Rasch R.F.R. (2019). Ancient History and New Frontiers: Infectious Diseases. Nurs. Clin. N. Am..

[2] Damme W., Lerberghe W. (2000). Editorial: Epidemics and Fear. Trop. Med. Int. Health.

[3] Heitzman J. (2020). Impact of COVID-19 Pandemic on Mental Health. Psychiatr. Pol..

[4] Super S., Pijpker R., Polhuis K. (2021). The Relationship between Individual, Social and National Coping Resources and Mental Health during the COVID-19 Pandemic in the Netherlands. Health Psychol. Rep..

[5] Pijpker R., van der Kamp D., Vader S., den Broeder L., Wagemakers A. (2022). Socioeconomic Status and Mental Health during the COVID-19 Crisis: Are Sense of Coherence, Sense of Community Coherence and Sense of National Coherence Predictors for Mental Health?. Health Psychol. Rep..

[6] Golińska P.B., Cieślak M., Hubert O., Bidzan M. (2021). Mental Health and the Symptoms of PTSD in People with Depression and Anxiety Disorders during the COVID-19 Pandemic. Int. J. Environ. Res. Public Health.

[7] Dahiya A. (2020). The Phenomenology of Contagion. J. Bioethical Inq..

[8] Taylor S.E., Stanton A.L. (2007). Coping Resources, Coping Processes, and Mental Health. Annu. Rev. Clin. Psychol..

[9] Eichenberg C., Grossfurthner M., Andrich J., Hübner L., Kietaibl S., Holocher-Benetka S. (2021). The Relationship Between the Implementation of Statutory Preventative Measures, Perceived Susceptibility of COVID-19, and Personality Traits in the Initial Stage of Corona-Related Lockdown: A German and Austrian Population Online Survey. Front. Psychiatry.

[10] Carver C.S., Scheier M.F., Segerstrom S.C. (2010). Optimism. Clin. Psychol. Rev..

[11] Alarcon G.M., Bowling N.A., Khazon S. (2013). Great Expectations: A Meta-Analytic Examination of Optimism and Hope. Pers. Individ. Differ..

[12] Benzel E. (2020). Optimism Verses Pessimism: The Choice Is Yours. World Neurosurg..

[13] Bidzan-Bluma I., Bidzan M., Jurek P., Bidzan L., Knietzsch J., Stueck M., Bidzan M. (2020). A Polish and German Population Study of Quality of Life, Well-Being, and Life Satisfaction in Older Adults During the COVID-19 Pandemic. Front. Psychiatry.

[14] Stueck M. (2021). The Pandemic Management Theory. COVID-19 and Biocentric Development. Health Psychol. Rep..

[15] Scott A.M., Dworkin I., Dukas R. (2018). Sociability in Fruit Flies: Genetic Variation, Heritability and Plasticity. Behav. Genet..

[16] Ng D.X., Lin P.K.F., Marsh N.V., Chan K.Q., Ramsay J.E. (2021). Associations Between Openness Facets, Prejudice, and Tolerance: A Scoping Review With Meta-Analysis. Front. Psychol..

[17] Woo S.E., Chernyshenko O.S., Longley A., Zhang Z.X., Chiu C.Y., Stark S.E. (2014). Openness to Experience: Its Lower Level Structure, Measurement, and Cross-Cultural Equivalence. J. Pers. Assess..

[18] Luo P., LaPalme M.L., Cipriano C., Brackett M.A. (2022). The Association Between Sociability and COVID-19 Pandemic Stress. Front. Psychol..

[19] Grabowski J., Stepien J., Waszak P., Michalski T., Meloni R., Grabkowska M., Macul A., Rojek J., Lorettu L., Sagan I. (2021). Social Isolation During COVID-19 Pandemic. Perceived Stress and Containment Measures Compliance Among Polish and Italian Residents. Front. Psychol..

[20] Michalski T., Brosz M., Stepien J., Biernacka K., Blaszczyk M., Grabowski J. (2021). Perceived Stress Levels among Ukrainian Migrant and LGBT+ Minorities in Poland during the COVID-19 Pandemic. Int. J. Environ. Res. Public Health.

[21] Xin M., Luo S., She R., Yu Y., Wang S., Tao F., Zhao J., Hu D., Gu J., Wang H. (2020). Negative Cognitive and Psychological Correlates of Mandatory Quarantine during the Initial COVID-19 Outbreak in China. Am. Psychol..

[22] Tang F., Liang J., Zhang H., Kelifa M.M., He Q., Wang P. (2021). COVID-19 Related Depression and Anxiety among Quarantined Respondents. Psychol. Health.

[23] Chu I.Y.H., Alam P., Larson H.J., Lin L. (2020). Social Consequences of Mass Quarantine during Epidemics: A Systematic Review with Implications for the COVID-19 Response. J. Travel Med..

[24] Lorettu L., Mastrangelo G., Stepien J., Grabowski J., Meloni R., Piu D., Michalski T., Waszak P.M., Bellizzi S., Cegolon L. (2021). Attitudes and Perceptions of Health Protection Measures Against the Spread of COVID-19 in Italy and Poland. Front. Psychol..

[25] Aschwanden D., Strickhouser J.E., Sesker A.A., Lee J.H., Luchetti M., Stephan Y., Sutin A.R., Terracciano A. (2021). Psychological and Behavioural Responses to Coronavirus Disease 2019: The Role of Personality. Eur. J. Pers..

[26] Jimenez T., Restar A., Helm P.J., Cross R.I., Barath D., Arndt J. (2020). Fatalism in the Context of COVID-19: Perceiving Coronavirus as a Death Sentence Predicts Reluctance to Perform Recommended Preventive Behaviors. SSM-Popul. Health.

[27] Mojzisch A., Elster C., Germar M. (2021). People Perceive Themselves to Adhere More Strictly to COVID-19 Guidelines than Others. Psychol. Health Med..

[28] Morales-Vives F., Dueñas J.M., Vigil-Colet A., Camarero-Figuerola M. (2020). Psychological Variables Related to Adaptation to the COVID-19 Lockdown in Spain. Front. Psychol..

[29] Valenti G.D., Faraci P. (2021). Identifying Predictive Factors in Compliance with the COVID-19 Containment Measures: A Mediation Analysis. Psychol. Res. Behav. Manag..

[30] Abdelrahman M. (2020). Personality Traits, Risk Perception, and Protective Behaviors of Arab Residents of Qatar During the COVID-19 Pandemic. Int. J. Ment. Health Addict..

[31] Rosenstock I.M. (1966). Why People Use Health Services. Milbank Mem. Fund Q..

[32] Stępień J., Michalski T., Grabowski J., Waszak P., Grabkowska M., Macul A., Rojek J.J. (2021). Social Response and Spatial Mobility Change Due to Covid-19 Pandemic in Poland. Geogr. Pol..

[33] Data on Country Response Measures to COVID-19. https://www.ecdc.europa.eu/en/publications-data/download-data-response-measures-covid-19.

[34] Mo P.K.H., Luo S., Wang S., Zhao J., Zhang G., Li L., Li L., Xie L., Lau J.T.F. (2021). Intention to Receive the Covid-19 Vaccination in China: Application of the Diffusion of Innovations Theory and the Moderating Role of Openness to Experience. Vaccines.

[35] Onyeaka H., Anumudu C.K., Al-Sharify Z.T., Egele-Godswill E., Mbaegbu P. (2021). COVID-19 Pandemic: A Review of the Global Lockdown and Its Far-Reaching Effects. Sci. Prog..

[36] Likert R. (1932). A Technique for the Measurement of Attitudes. Arch. Psychol..

[37] Webster R.K., Brooks S.K., Smith L.E., Woodland L., Wessely S., Rubin G.J. (2020). How to Improve Adherence with Quarantine: Rapid Review of the Evidence. Public Health.

[38] Hingle M.D., Wertheim B.C., Tindle H.A., Tinker L., Seguin R.A., Rosal M.C., Thomson C.A. (2014). Optimism and Diet Quality in the Women’s Health Initiative. J. Acad. Nutr. Diet..

[39] Tindle H., Belnap B.H., Houck P.R., Mazumdar S., Scheier M.F., Matthews K.A., He F., Rollman B.L. (2012). Optimism, Response to Treatment of Depression, and Rehospitalization after Coronary Artery Bypass Graft Surgery. Psychosom. Med..

[40] Potempa K. (2013). Optymizm a Zdrowie. Med. Ogólna I Nauk. O Zdrowiu.

[41] Abramson L.Y., Seligman M.E., Teasdale J.D. (1978). Learned Helplessness in Humans: Critique and Reformulation. J. Abnorm. Psychol..

[42] Woodland L., Hodson A., Webster R.K., Amlôt R., Smith L.E., Rubin J. (2022). A Qualitative Study Evaluating the Factors Affecting Families’ Adherence to the First COVID-19 Lockdown in England Using the COM-B Model and TDF. Int. J. Environ. Res. Public Health.

[43] Smith L.E., Amlȏt R., Lambert H., Oliver I., Robin C., Yardley L., Rubin G.J. (2020). Factors Associated with Adherence to Self-Isolation and Lockdown Measures in the UK: A Cross-Sectional Survey. Public Health.

[44] Pollak Y., Dayan H., Shoham R., Berger I. (2020). Predictors of Non-Adherence to Public Health Instructions during the COVID-19 Pandemic. Psychiatry Clin. Neurosci..

[45] Thunander Sundbom L., Bingefors K. (2012). Hombres y Mujeres Comunican Diferentes Comportamientos y Razones Para El Incumplimiento de La Medicación: Encuesta Nacional Sueca. Pharm. Pract. (Granada)..

[46] Feldman G., Chao M.M., Farh J.L., Bardi A. (2015). The Motivation and Inhibition of Breaking the Rules: Personal Values Structures Predict Unethicality. J. Res. Pers..

[47] World Health Organization Coronavirus Disease (COVID-19): Health and Safety in the Workplace. https://www.who.int/news-room/questions-and-answers/item/coronavirus-disease-covid-19-health-and-safety-in-the-workplace.

[48] Forman D.E., Maurer M.S., Boyd C., Brindis R., Salive M.E., Horne F.M.F., Bell S.P., Fulmer T., Reuben D.B., Zieman S. (2018). Multimorbidity in Older Adults With Cardiovascular Disease. J. Am. Coll. Cardiol..

[49] Ackermann M., Verleden S.E., Kuehnel M., Haverich A., Welte T., Laenger F., Vanstapel A., Werlein C., Stark H., Tzankov A. (2020). Pulmonary Vascular Endothelialitis, Thrombosis, and Angiogenesis in Covid-19. N. Engl. J. Med..

[50] Bogg T., Milad E. (2020). Demographic, Personality, and Social Cognition Correlates of Coronavirus Guideline Adherence in a U.S. Sample. Health Psychol..

[51] Choi S.L., Martin P., Cho J., Ryou Y.J., Heinz M. (2022). Personality and Compliance with COVID-19 Protective Measures among Older Americans: Moderating Effects of Age, Gender, and Race/Ethnicity. Pers. Individ. Differ..

[52] Willroth E.C., Smith A.M., Shallcross A.J., Graham E.K., Mroczek D.K., Ford B.Q. (2021). The Health Behavior Model of Personality in the Context of a Public Health Crisis. Psychosom. Med..

[53] Starcevic V., Janca A. (2022). Personality Dimensions and Disorders and Coping with the COVID-19 Pandemic. Curr. Opin. Psychiatry.

[54] McColl K., Debin M., Souty C., Guerrisi C., Turbelin C., Falchi A., Bonmarin I., Paolotti D., Obi C., Duggan J. (2022). Are People Optimistically Biased about the Risk of Covid-19 Infection? Lessons from the First Wave of the Pandemic in Europe. Int. J. Environ. Res. Public Health.

[55] Fekadu G., Bekele F., Tolossa T., Fetensa G., Turi E., Getachew M., Abdisa E., Assefa L., Afeta M., Demisew W. (2021). Impact of COVID-19 Pandemic on Chronic Diseases Care Follow-up and Current Perspectives in Low Resource Settings: A Narrative Review. Int. J. Physiol. Pathophysiol. Pharmacol..

[56] Wnuk A., Oleksy T., Maison D. (2020). The Acceptance of Covid-19 Tracking Technologies: The Role of Perceived Threat, Lack of Control, and Ideological Beliefs. PLoS ONE.

[57] Tseng C.-W.W., Roh Y., DeJong C., Kanagusuku L.N., Soin K.S. (2021). Patients’ Compliance With Quarantine Requirements for Exposure or Potential Symptoms of COVID-19. Hawai’i J. Health Soc. Welf..

[58] Krumpal I. (2013). Determinants of Social Desirability Bias in Sensitive Surveys: A Literature Review. Qual. Quant..

[59] Boswell N., Cao J., Torres W.J., Beier M., Sabharwal A., Moukaddam N. (2020). A Review and Preview of Developments in the Measurement of Sociability. Bull. Menn. Clin..

