# Supplementary material for: Self-Assessed Personality Traits and Adherence to the COVID-19 Lockdown
Source: Int J Environ Res Public Health. 2022 Dec 28;20(1):521. doi: 10.3390/ijerph20010521 (PMC9819452; doi:10.3390/ijerph20010521)
Supplement: Supplementary file 1 [file ijerph-20-00521-s001.zip › ijerph-2018363-supplementary.pdf]

Variable	Raw Alpha	Change
Self-assesed personality traits		
Sociability	0.443416	-0.166738
Calmness	0.724527	0.114373
Openness	0.483625	-0.126529
Optimism	0.440306	-0.169849
Lockdown compliance		
I occasionally do some essential grocery shopping (1-2 times a week)	0.703044	0.034182
I go out primarily to work	0.709127	0.040265
I go out for a walk in my neighborhood	0.618789	-0.050074
I go out for a walk to public places (parks, beaches, boulevard etc.)	0.598551	-0.070311
I do sports outside (running, cycling etc)	0.588475	-0.080387
I regularly go to church/mosque/temple/place of religious worship	0.616544	-0.052319
I regularly meet with friends	0.615274	-0.053588
I leave the house only in the most needed matters	0.661151	-0.007711
Everyday I go out with my child/pet	0.638641	-0.030221
